# Detecting structural variances of Co_3_O_4_ catalysts by controlling beam-induced sample alterations in the vacuum of a transmission electron microscope

**DOI:** 10.1186/s40679-016-0027-9

**Published:** 2016-11-02

**Authors:** C. Kisielowski, H. Frei, P. Specht, I. D. Sharp, J. A. Haber, S. Helveg

**Affiliations:** 1 Molecular Foundry, Lawrence Berkeley National Laboratory, 1 Cyclotron Road, Berkeley, CA 94720 USA; 2 Physical Biosciences Division, Lawrence Berkeley National Laboratory, 1 Cyclotron Road, Berkeley, CA 94720 USA; 3 Department of Material Science and Engineering, University of California–Berkeley, Berkeley, CA 94720 USA; 4 Chemical Sciences Division and Joint Center for Artificial Photosynthesis, Lawrence Berkeley National Laboratory, Berkeley, CA 94720 USA; 5 Joint Center for Artificial Photosynthesis California Institute of Technology, Pasadena, CA 91125 USA; 6 Haldor Topsoe A/S, Haldor Topsøes Allé 1, 2800 Kongens Lyngby, Denmark

## Abstract

This article summarizes core aspects of beam-sample interactions in research that aims at exploiting the ability to detect single atoms at atomic resolution by mid-voltage transmission electron microscopy. Investigating the atomic structure of catalytic Co_3_O_4_ nanocrystals underscores how indispensable it is to rigorously control electron dose rates and total doses to understand native material properties on this scale. We apply in-line holography with variable dose rates to achieve this goal. Genuine object structures can be maintained if dose rates below ~100 e/Å^2^s are used and the contrast required for detection of single atoms is generated by capturing large image series. Threshold doses for the detection of single atoms are estimated. An increase of electron dose rates and total doses to common values for high resolution imaging of solids stimulates object excitations that restructure surfaces, interfaces, and defects and cause grain reorientation or growth. We observe a variety of previously unknown atom configurations in surface proximity of the Co_3_O_4_ spinel structure. These are hidden behind broadened diffraction patterns in reciprocal space but become visible in real space by solving the phase problem. An exposure of the Co_3_O_4_ spinel structure to water vapor or other gases induces drastic structure alterations that can be captured in this manner.

## Introduction

### Background

Since the advent of high voltage electron microscopy, electron beam-induced damage in the bulk of crystalline materials has been extensively studied (e.g., [1]). In the traditional picture, it is understood that the dominant interaction of the electron beam with the sample causes atom displacements by knock-on events. Remarkably, the rapidly improving performance of electron microscopes operating in the mid-voltage range, between 20 and 300 kV [2–5], has made it possible to obtain atomic resolution images with single atom sensitivity at voltages well below the abrupt threshold values for atom displacements from their bulk lattice sites. Therefore, one is tempted to speculate that atom displacements are not longer of concern. Indeed, recent experimental investigations show that bulk materials can stand long time periods of electron irradiation before any beam-induced sample alteration is recognized [6]. However, it would be inaccurate to conclude that electron beam-induced damage is now absent while observing the atomic structure of materials. Indeed, unobtrusive damage processes remain very active in the mid-voltage range. In particular, concerns arise in investigations that aim at detecting single atoms because the electron dose needed to create sufficient contrast is very large. Importantly, scientifically attractive investigations fall into this category. For example, atomic-resolution electron tomography [7–10], the imaging of single molecules [11], and the determination of structure–function relationships in catalytic processes [12, 13] all demand resolution and sensitivity down to the level of single atoms. However, it will be pointed out in this paper that the detection of single atoms requires the application of electron doses as large as 500–10,000 e/Å^2^ that are often delivered within 1 s during image acquisition while—at the same time—it becomes evident that electron dose rates above ~100 e/Å^2^s can already be too large if it is attempted to maintain structural integrity of small catalytic metal oxide electrocatalysts, for example [12]. This obvious gap between tolerable and needed electron dose (or rate) to detect single atoms is addressed in this contribution.

Advanced structural and chemical imaging with atomic resolution relies on the extraordinary performance of aberration-corrected electron microscopes that are equipped with high-brightness electron sources. These are required to provide the large beam currents (dose rates) [2, 5, 14] needed to generate interpretable contrast from electron scattering at single atoms. The total electron dose must be large because such scattering events contribute very little contrast to atomic resolution images from crystals, which are typically dominated by the much larger contrast of atom columns. However, the atomic structures of defects, interfaces, and surfaces are all preferentially affected by beam-induced object alterations because the binding energies of relevant atoms are significantly lowered compared to those of bulk lattice sites in crystalline solids. As a consequence, unintentional sample alterations are often obscured by a lack of sensitivity, the choice of forgiving detection modes, the absence of time resolution, and other factors [16], such that they are easily overlooked and may trigger misleading conclusions. Consequently, a variety of strategies are now deployed [3, 5, 15, 17, 18] that aim at overcoming the various aspects of electron beam-induced sample alterations at the single atom level in atomic resolution electron microscopy.

As a guideline, one must expect that relevant object distortions will occur during investigations of nanostructured soft and hard matter, where a rich variety of defects, interfaces, and surfaces allows forging new material properties that rapidly emerge worldwide. Furthermore, in environmental electron microscopy, liquids and gases are easily ionized along the path of the electron beam [19], which can lead to unexpected interactions of materials with their surroundings. This paper utilizes Co_3_O_4_ catalysts as an example to summarize emerging capabilities that take advantage of controlling electron doses and rates to understand how crystal structures can be maintained or altered in a controlled fashion.

### A summary of beam-induced sample alterations

**Fig. 1:**
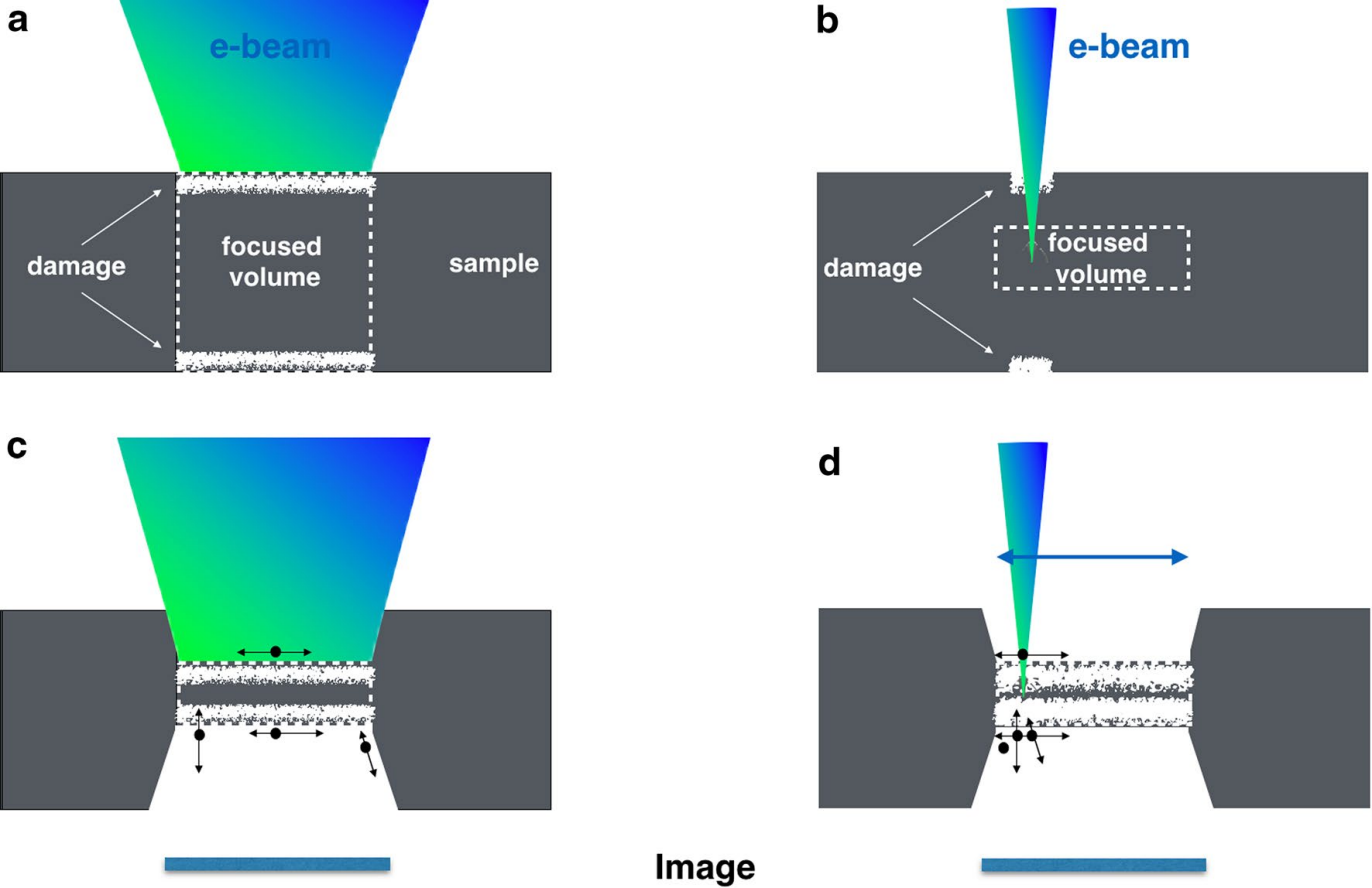
Schematics of electron beam-induced object alterations in the mid-voltage range. Damaged areas are dominantly generated in surface proximity and symbolized by wide diffuse lines. HRTEM (**a**, **c**) and STEM (**b**, **d**) detection modes are considered. **a**, **b** Initial situation for thick samples. **c**, **d** Structure after a prolonged exposure to the electron beam (e-beam) or thin sample. The ongoing sputtering process removes atoms (*black dots*) from the *top* and *bottom* surfaces at different rates. In HRTEM mode, the beam is static, the semi-convergence angle is ~0.1 mrad and the dose rate can be varied between 1 atto A/Å^2^ and 1 pico A/Å^2^. In STEM mode, the beam is scanned, the semi-convergence varies between 5 and 25 mrad and the dose rate typically changes between 1 and 100 pico A/Å^2^. The illuminated area is matched to the imaged area (camera). For more details see text

Figure 1 illustrates how samples are altered by accelerated mid-voltage electrons in broad beam high resolution transmission electron microscopy (HRTEM) and in focused beam scanning transmission electron microscopy (STEM). A situation is depicted in which the illuminated sample area matches the imaged sample area. In HRTEM, this match is achieved by a Nelsonian illumination scheme [20] (Fig. 1a), and in STEM (Fig. 1b), it is naturally set by the scanning range. In this case, both detection modes can be compared. It is well established that electrons from the mid-voltage range preferentially remove atoms in surface proximity [21–23]. However, unlike the abrupt onset of knock-on damage in bulk materials, this onset is continuous and occurs at much lower energies because of the distribution of reduced binding energies of atoms at surfaces, interfaces, or defects [24, 25]. In fact, surface effects entirely dominate if the sample dimensions shrink to the range of single-digit nanoparticles, where entire particles can become unstable in the electron beam [20, 26].

In Fig. 1, altered sample surfaces are symbolized by irregular white bands. An image of a thick sample acquired in broad beam mode (Fig. 1a) typically captures the entire illuminated sample volume, including bulk and surface contributions. Current densities can be chosen as small as 1 atto Ampere per square Ångstrom (aA/Å^2)^, which delivers only six electrons to each square Ångstrom of the sample in a second (e/Å^2^s). Such minimal current densities are typically utilized to capture images in cryo-microscopy because they prevent radiation-sensitive organic tissue or molecules from degrading during their observation [27, 28]. In contrast, a cone shaped STEM beam produces images of thickness slices from the sample. Conditions can be chosen to capture the interior of samples, thereby showing a pattern that is only marginally distorted by blurred, unfocused contrast contributions from the sample surfaces [14], as depicted in Fig. 1b. As a result of the cone shaped illumination, the current density at the sample surface can be smaller compared to its value in the focused beam spot and surface damage can be reduced. For example, a 50 pA probe, with a beam semi-convergence angle of 25 mrad that is focused 100 Å deep into the sample to form a spot of 1Å diameter, would spread over an area of ~20 Å^2^ at the crystal surface. Thus, the current density at the sample surface is reduced to ~2.5 pA/Å^2^, which is equivalent to 1.5 10^7^ e/Å^2^s. Buban et al. [3] matched the total electron dose of STEM experiments to the needs of cryo-microscopy by choosing a ~1 pA beam current, in combination with a short dwell time of ~1 μs. Both the blurred contrast contributions from surfaces—if present—and the selective amplification of contrast from heavy atoms can make high angle annular dark field (HAADF) STEM images appear more stable than their TEM counterparts, but at the cost of a reduced information content concerning sample thickness and strain contrasts (e.g., dislocations). Upon prolonged beam exposure, however, the unavoidable sputtering of surface atoms reduces the sample thickness such that both techniques simultaneously capture bulk and surface contributions in single images (Fig. 1c, d). In any case, electron microscopy certainly allows for a damage-free acquisition of images even from organic matter if the total electron dose is kept below ~20 e/Å^2^ [1, 3, 4] and contrast remains sufficiently high, which is easily achievable in the low magnification range.

**Fig. 2:**
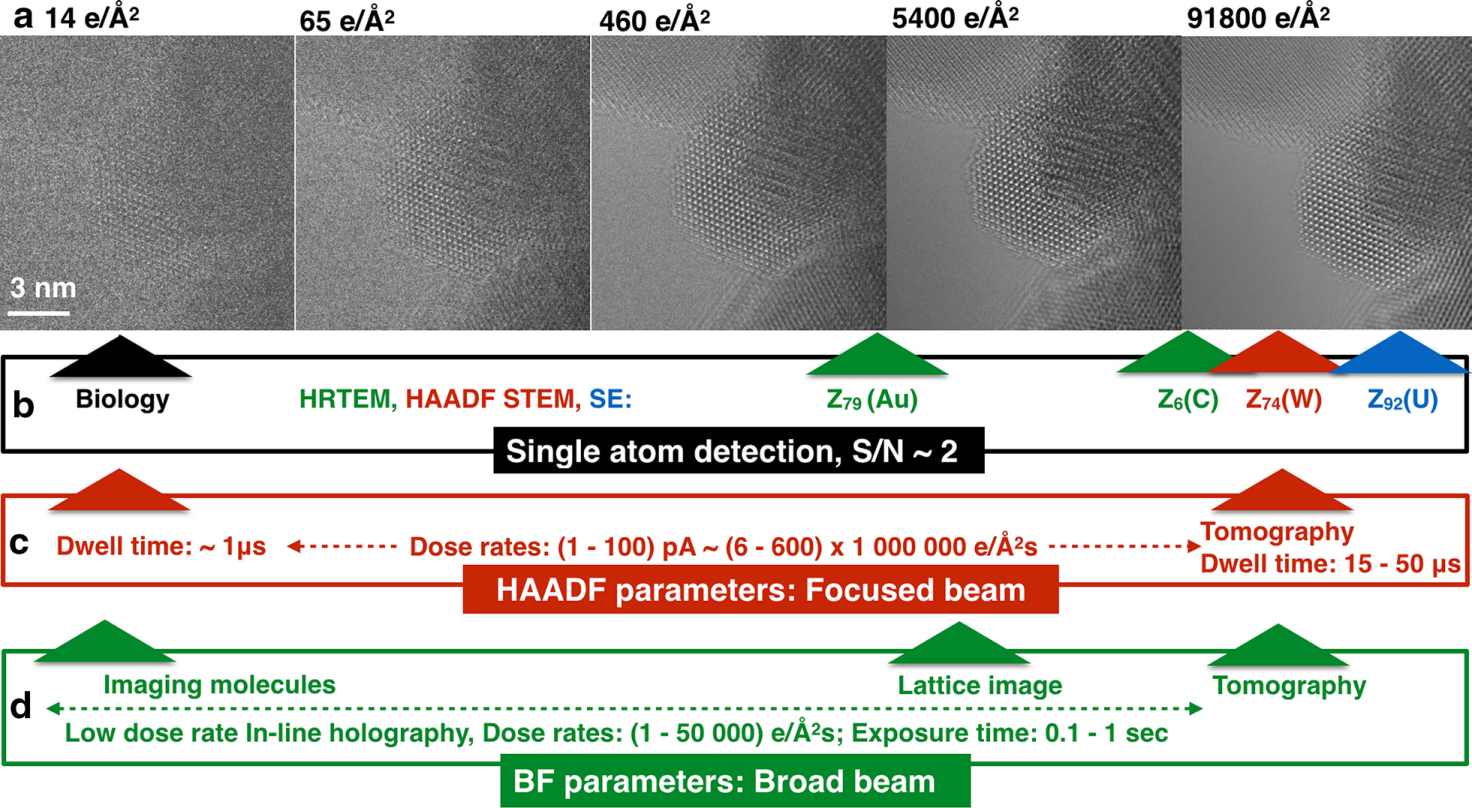
Dose dependent contrast and single atom detection. **a** Single images of a spinel Co_3_O_4_ [114] catalyst recorded with the indicated total electron dose per image. A 1 s exposure time was used. **b** Estimated total electron doses for the detection of single atoms with different atomic number Z in BF HRTEM (*green*), HAADF STEM (*red*), and SE (*blue*) detection modes. For details see text. **c** Typical dose rates and time constants in HAADF STEM mode. **d** Typical dose rates and time constants in HRTEM mode. Low dose rate in-line holography [4] can be used to record images with the low dose rates that are used in biological sciences and the method allows to build up the needed total dose for the detection of single atoms by the acquisition of large image series

Concerns regarding beam-induced sample alterations arise if it is attempted to understand structure and chemical composition of materials at atomic resolution with single atom sensitivity. For example, Fig. 2a shows atomic resolution HRTEM images of a ~5 nm sized Co_3_O_4_ catalyst imaged along its [114] zone axis orientation with total electron doses between ~10 and ~100,000 e/Å^2^. Bright spots in the images mark the location of atom columns that are detectable in all images but signal-to-noise (S/N) ratios naturally increase with the total electron dose. Certainly, it is not obvious from the images how many electrons are needed to reliably identify scattering contributions from single atoms.

S/N ratios for the detection of single atoms can be estimated from the literature and their magnitude is indicated in Fig. 2b). For the aberration-corrected TEAM0.5 microscope, it was reported that a single gold atom with its atomic number Z of 79 (Z_79_) can be detected in HRTEM images with a S/N ratio of 2 [14], if a total electron dose of ~2300 e/Å^2^ is applied at 300 kV. A voltage reduction to 80 kV improves on contrast by a factor of ~2 [29], which allows shot noise (N/**√**N) to be decreased by a factor of 4 to maintain the same S/N ratio. Therefore, the total electron dose for the detection of a single gold atom at 80 kV decreases to ~575 e/Å^2^. Single carbon atoms in graphene are typically observed with a S/N ratio of 2 at 80 kV using a total dose of ~20,000 e/Å^2^ [30]. Ignoring contributions from inelastic scattering, this dose difference for the detection of single carbon and gold atoms with S/N ratios around 2 can quantitatively be expected because electron scattering depends on the atomic number (Z); it obeys a Z^0.66^ rule in the bright field (BF) HRTEM mode [31] and a Z^1.7^ rule in the STEM high angle annular dark field (HAADF) mode [32]. In HAADF STEM at 300 kV, the contrast from single, heavy atoms can be reliably identified from its contribution to the contrast of atom columns [8]. We measured S/N ratios between 2 and 4 at 300 kV for the detection of a single gold atom at a resolution between 0.5 and 1 Å for total electron doses between 35,000 and 70,000 e/Å^2^ in single HAADF images. Similar doses are applied to single images to produce tomograms of tungsten atoms in three dimensions.^1^ Certainly, these estimates cannot be more accurate than a factor of 2 or 3 but they provide very useful guidance for choosing total doses required for different experiments, as shown in this paper.

Thus, the current technology allows detection of single heavy atoms with comparable S/N ratios at 300 kV if the total electron dose in HAADF STEM images exceeds the BF HRTEM dose by a factor of roughly 20. This difference originates from the less effective detection of only those electrons that are scattered to large angles in reciprocal space, where all information is lost about the real space localization of the scattering events. S/N ratios diverge even further if light atoms are considered because of the distinctly different Z-dependences of electron scattering in the HAADF and BF imaging modes. Single atoms can also be detected at atomic resolution by collecting secondary electrons (SE) [33]. However, in this case, the S/N ratio for the detection of a single uranium atom is even poorer than the contrast of a single uranium atom recorded in an annular dark field STEM mode.

Given the considerations discussed above, it is not surprising that it has become common to improve on the STEM detection efficiency by reducing the acceleration voltage and the inner angle of the dark field detector to capture more electrons [2] or by directly using BF detectors [34]. Nevertheless, the real space HRTEM mode remains not only the most effective detection scheme but also the fastest way to capture images of scattered electrons because of the parallel electron detection. Consequently, it is used in biological [28] and environmental sciences [35], where obtaining the largest contrast with the fewest number of primary electrons possible matters greatly.

Time constants differ greatly in both detection modes, since aberration-corrected HAADF STEM and BF HRTEM imaging modes build on distinctly different technologies. Typical parameter ranges are given in (Fig. 2c, d), respectively. In STEM, the relevant parameters are the local beam current and the dwell time, which set the total electron dose for probes that, can be focused to the diameter of a single atom column. With current equipment, beam currents of (6–600) × 10^6^ e/Å^2^s are used and total doses are set by dwell times ranging from microseconds to milliseconds. Low dose images of radiation-sensitive matter can be obtained by combining a beam current around 1 pA with a dwell time of 1 μs to produce images of crystalline solids that are largely noise dominated [3]. In the other extreme, typical dwell times of 15–50 μs are used in combination with beam currents of approximately 50 pA to enable electron tomography with single, heavy atom sensitivity and atomic resolution. In the HRTEM mode (Fig. 2d), typical image exposure times of 0.1–1 s and dose rates between 1 and 50,000 e/Å^2^s determine the total dose. Thus, time constants and dose rates differ roughly by up to six orders of magnitude if STEM and HRTEM modes are compared.

Further differences between the detection modes are caused by the convergence of the electron beam to form a local probe, which forces a serial image acquisition of thickness slices with an increased spread of the electron beam in z-direction because of electron channeling in zone axis orientations of the samples. Further, the detection of electrons in real space is most effective. For convenience, the Table 1 summarizes relevant differences between broad beam and focused probe detection modes.

**Table 1:** A summary of relevant differences between imaging in broad beam mode and with a focused electron beam

Relevant differences	Broad beam (preferred in biology)	Focused beam
Dose rates	1–10 000 e/Å^2^s	(6–600) * 1,000,000 e/Å^2^s
Time constants (image acquisition)	0.1–10 s	1–100 μs per pixel
Minimal spread in beam direction (highest resolution)	<7 Å	~40 Å
Electron detection	Real space	Reciprocal space
Acquisition process	Parallel	Serial
Visibility	Entire sample thickness	Thickness slices (resolution dependent)

It was recently demonstrated that electron beam-induced sample alterations can be effectively retarded by recording images with low dose rates and accumulating the needed total electron dose by acquiring image series that contain hundreds of frames [26]. Our implementation consists of retrieving exit wave functions form focus series of images to obtain in-line holograms that always capture the whole elastic scattering information unlike single images. In doing so, the contrast transfer function (CTF) starts oscillating with increasing defocus. Typically, we balance the third-order spherical aberration C_3_ with the fifth-order spherical aberration C_5_ to maximize contrast transfer close to 1 [36] and capture all elastically scattered electrons. With increasing defocus, CTF oscillations occur and half of all electrons contribute to the contrast formation since the average of a squared sinusoidal oscillation equals 1/2. In addition, the range of defocus values can be suitably adjusted to extend the low frequency limit to 4–5 nm if needed.

In this manner, best practices known from biological sciences are adopted for the acquisition of atomic resolution images [27]. Finally, the low-dose rate approach also allows for self-consistent electron tomography from single projections, which preserves pristine atomic structures most effectively [10].

Initially, electron beam-induced object alterations only cause small contrast changes in images since they relate to the loss of single atoms from the much stronger contrast of atom columns. Thus, they are hardly recognizable by visual inspection. Only the accumulation of damage can be readily observed. Therefore, a quantitative contrast analysis is not optional but mandatory, if genuine structure of surfaces, defects, or other radiation-sensitive matter, including catalysts is to be interpreted. As a minimum requirement, it should become good practice to list electron doses and rates with every published image. This paper presents detailed examples that highlight the pressing need to address beam-sample interactions in investigations of catalytic nanocrystals using the BF HRTEM mode, where primary beam currents and total doses can be tuned very effectively. The derived conclusions, however, are equally valid for broad beam and focused probe imaging modes and can be converted using the guidelines of Fig. 2.

### Experimental details

The investigated Co_3_O_4_ samples (space group 227, volume = 525 Å^3^, 56 atoms in the unit cell) are prepared by three different methods:

- A high throughput inkjet printing process of the (Ni–Fe–Co–Ce)O_x_ composition space for active OER electrocatalysts [12], which includes a successive calcination step at 350–400 °C.
- A surfactant-assisted solvothermal method, as previously reported by Agiral et al. [37].
- A plasma-assisted atomic layer deposition (ALD) method in which a thin polycrystalline film of CoO_x_ catalyst is deposited at a substrate temperature of 100 °C [38].

In all cases, the detected electron nano-diffraction patterns of the as-prepared samples can be described by the spinel phase of Co_3_O_4_ [39]. Different crystal structures were observed in surface proximity of the nanocrystals and after exposure of the material to gaseous environments at elevated temperature.

Low dose rate in-line electron holography is performed with the TEAM0.5 microscope, which can be operated between 20 and 300 kV [4, 15]. Focus series containing up to 100 images are recorded with variable dose rates and reconstructed with the McTempas software [41] to produce electron exit wave functions, which are in-line holograms. In this process, the phase problem is solved using a Gerchberg–Saxton algorithm [40]. The Fourier transforms of the complex electron exit wave functions provide all depicted nano-diffraction patterns. Unlike a Fourier transform of real images, they do not exhibit Friedel symmetry and allow measuring sample tilt. The software is also used for image analysis and multislice calculations [41]. Doses and rates are calculated from counts on the CCD camera, which are calibrated by a known number of electrons that are emitted from the filament for operating voltages of 80 and 300 kV.

The electron transparent samples are produced by crushing the fabricated powders and dispersing flakes of the Co_3_O_4_ catalysts onto grids for electron microscopy made from stainless steel. The ALD of the Co_3_O_4_ is performed directly onto an electron transparent SiN_x_ membrane [38].

An exposure of the samples to water vapor at a temperature of 400 °C is accomplished in an environmental electron microscope that is described in detail elsewhere [42].

## Results and discussion—beam-induced object alterations

### Pristine and altered Co_3_O_4_ surfaces

**Fig. 3:**
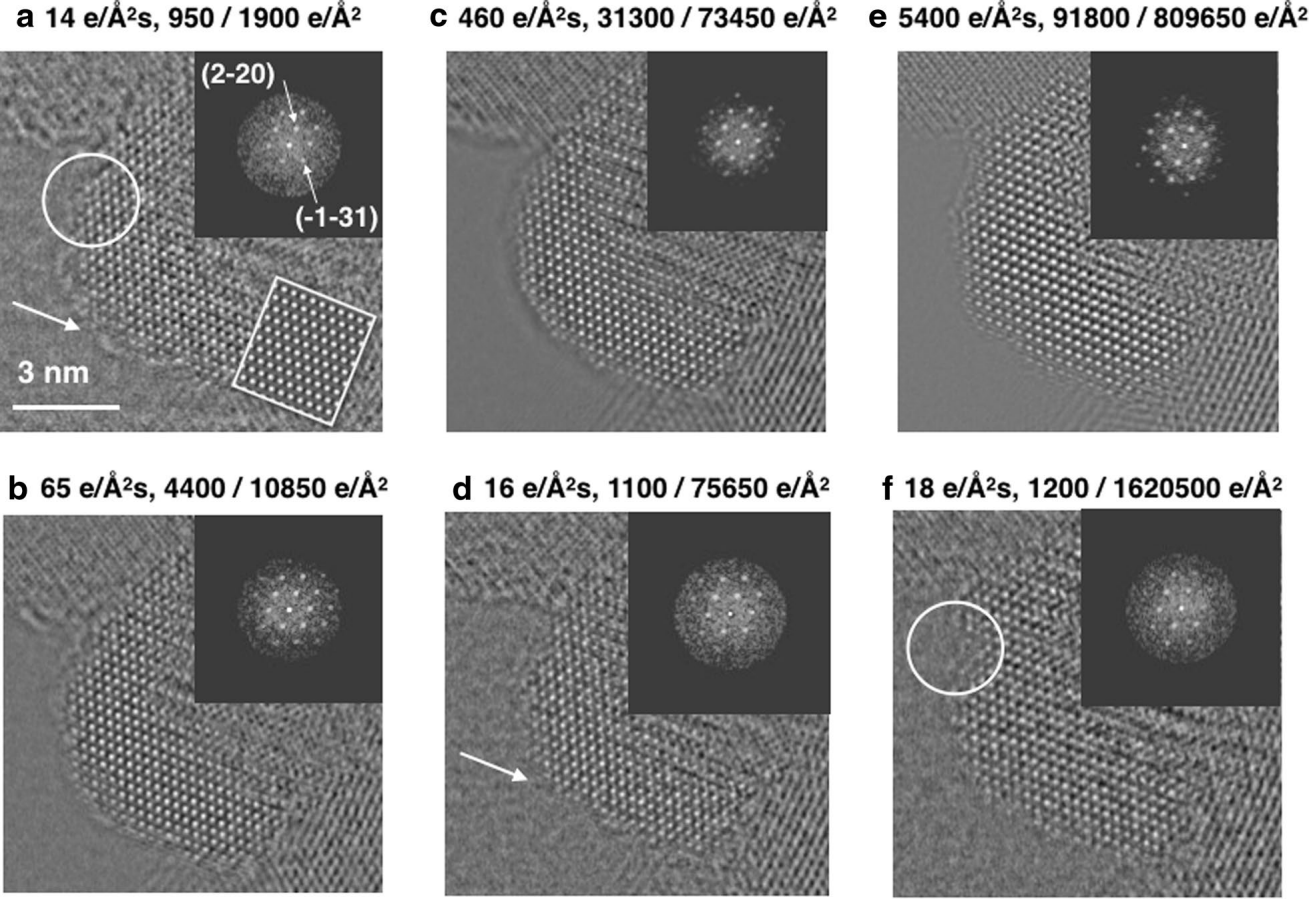
Dose dependent atom reconfigurations in surface proximity captured in sequential phase images. The mid-voltage range electron beam acceleration is 80 kV. A pristine spinel Co_3_O_4_ [114] particle is displayed in (**a**). Compared with the single images of Fig. 2a, its contrast is enhanced by reconstructing the complex electron exit wave functions from focus series of 40 images. Electron dose rates, the total dose per image series, and the accumulated electron dose are listed. Nano-diffraction patterns are shown in the insets. A simulated image pattern of spinel Co_3_O_4_ [114] is depicted in the *inset* of (**a**). The *arrows* and *circles* point to electron beam-induced surface alterations that occurred as a result of exposing the sample to an increasing number of electrons as listed. The sample was prepared by the high throughput printing process. **b**–**f** Are successive phase images of reconstructed wave functions that were recorded with the listed dose rates, total dose of the image series and accumulated electron dose

First, it is shown in Fig. 3 in which circumstances surface alterations occur. For this purpose, we consider phase images of the electron exit wave function reconstructed from focus series that include 50–100 image frames. Unlike single images, these reconstructed series reveal the crystal structure even if the structural information is overwhelmed by noise in the single low dose rate images that are shown in Fig. 2. Total and accumulated electron doses are chosen such that the reconstructed phase images of the focus series can be compared with usual acquisition parameters for HRTEM images. Relevant phase images are shown in Fig. 3a–c, which were recorded with 14, 65 and 460 e/Å^2^s, respectively. Figure 3d, f reproduce the experiment with dose rates similar to those of Fig. 3a, after applying a higher dose exposure, thereby allowing for a direct comparison of the irradiated material with the initial object structure. From subtle differences in Fig. 3a and d, it can be recognized that initially attached surface layers are removed from the catalyst and that its crystalline surfaces are restructured to a depth of one or two crystalline monolayers, as highlighted for a specific location by arrows. Thus, surface features can be lost without being noticed in single images, even if dose rates and total doses are moderate. Detecting the presence and genuine structure of such surface features is highly relevant for understanding the native catalytic surface activity. The inserted nano-diffraction patterns show minor changes of the crystal orientation that can become significant if the utilized dose rates are high enough to locally heat the samples [43].

A total electron dose of 950 e/Å^2^, such as used in Fig. 3a, would suffice to observe single heavy atoms, such as gold, with an S/N ratio around 2. A dose of 31,300 e/Å^2^, as used in Fig. 3c, would allow detection of single carbon atoms. Similarly, high total doses are used in emerging approaches to electron tomography: an estimated electron dose of 90,000 e/Å^2^ was used at 300 kV to record a single HRTEM image to reconstruct a tomogram of MgO with single light atom sensitivity.^2^ In another study, a dose of ~30,000 e/Å^2^ was used to capture individual HAADF images within a 62 frame image series to produce a tomographic reconstruction of a tungsten needle tip (Footnote 1). In this case, the accumulated electron dose approaches 2,000,000 e/Å^2^ see (Footnote 1). Figure 3d, f show that one must expect that unprotected surfaces are significantly compromised by such imaging conditions. Consequently, a tomographic reconstruction from image series acquired with high dose rates will include uncontrolled time averages of a dynamic situation that can be confused with the genuine structure of nanocrystals. Tomographic reconstructions from single projections, in combination with acquisition of low dose rate images, can address this issue [10].

**Fig. 4:**
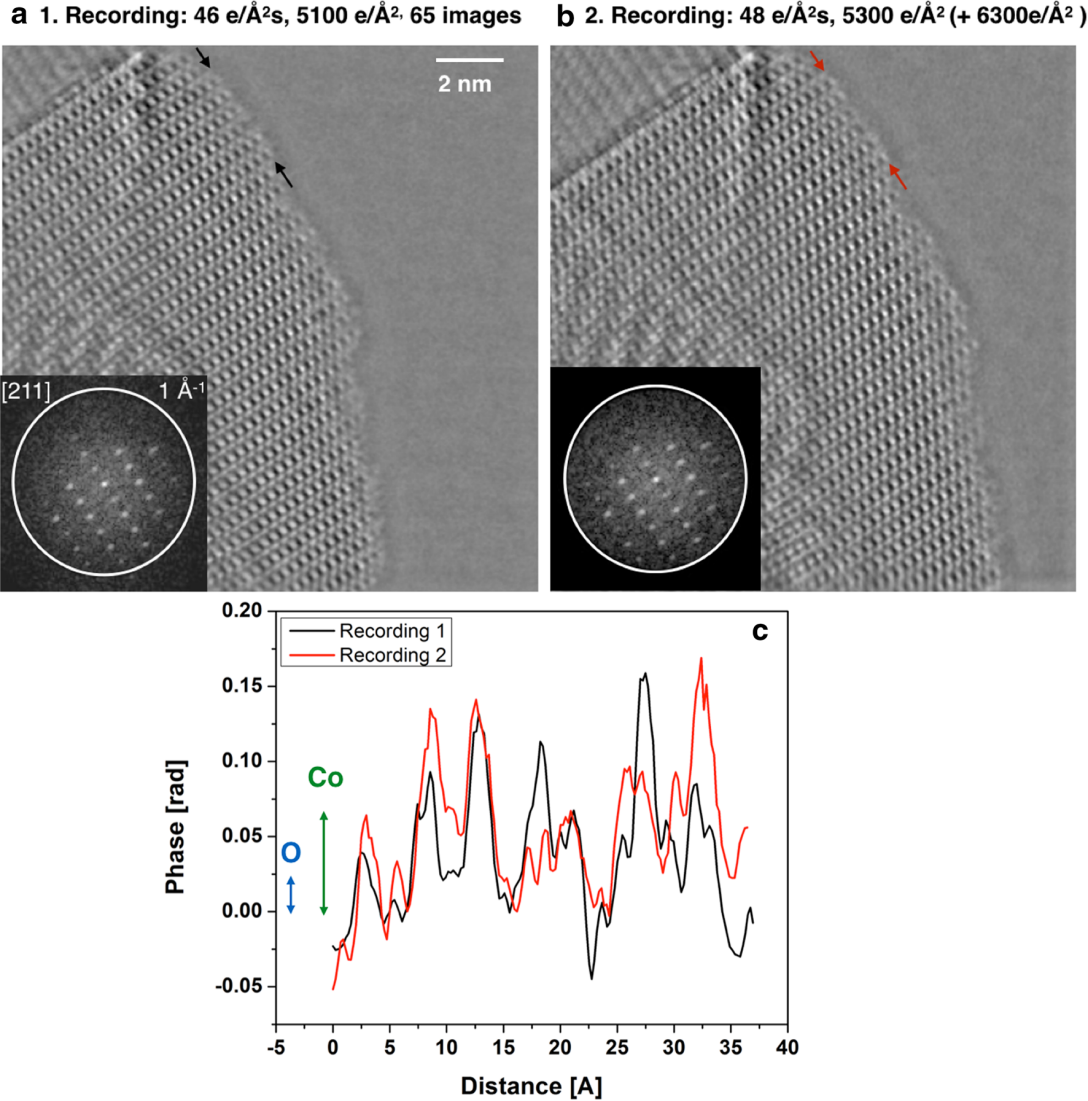
Sequential low dose-rate phase images **a**, **b** of a spinel Co_3_O_4_ particle. The mid-voltage range electron beam acceleration is 80 kV. A total of 160 images were recorded to produce the result. The sample was prepared by the high throughput printing process and exposed to technical air (N_2_, O_2_) at 400 °C for 2 h. The inserted nano-diffraction patterns show crystal tilt away from the [211] zone axis. Dose rate, total dose and accumulated dose are indicated and* arrows* mark the location of line traces that yield the contrast (phase) profiles of **c**. For comparison, expected phase changes from single O and Co atoms are indicated in **c**, too

Since our in-line holography approach retards beam-induced structure alterations, the question arises: to what measurable extent can structural integrity be maintained? Reconstructed phase images from two successively recorded low dose-rate image series are shown in Fig. 4a, b. Careful image inspection certainly suggests that the method faithfully reproduces the structure of the catalyst and its surfaces in great detail, and reveals that its initial surfaces are rough at the atomic scale if recorded in low dose-rate conditions. Faint contrast differences are present in the outermost layer of the catalyst at the Co_3_O_4_/vacuum interface and we quantify their magnitude by extracting line profiles from an identical area that is marked by arrows in both images. Line profiles are compared in Fig. 4c. It is seen that intensity maxima occur in identical locations but with variable intensity and on a fluctuating baseline. Comparing these fluctuations with the literature data for phase changes caused by electron scattering at single atoms [44] (inset in Fig. 4c), it is seen that the fluctuations are very small: they are comparable with the signal from a single oxygen atom, while the larger contrast of the heavier Co atoms can already be recognized with a S/N ratio of 1–2. Such faint contrasts reveal that the Co_3_O_4_/vacuum interface is composed of a layer that holds incomplete unit cells that contributes to surface roughness. Since the unit cell of Co_3_O_4_ is 8 Å large and contains 56 atoms, we probe for single atoms within a unit cell of Co_3_O_4_ and can infer if single cobalt atoms are kept in place or not to better understand the chemical composition of surfaces. A possible loss of single oxygen atoms, however, would hardly be recognized because a total dose of 5100 e/Å^2^ does not suffice to generate enough signals above noise to detect. In the TEAM0.5 microscope, a total dose of ~20,000 e/Å^2^ is needed for the detection of light atoms, which was not targeted in this experiment. Surely, such capabilities can be tuned to provide unique insight into atom dynamics and can be brought to good use in diverse applications.

### Structural Co_3_O_4_ alterations by electron beam-stimulated atom diffusion

**Fig. 5:**
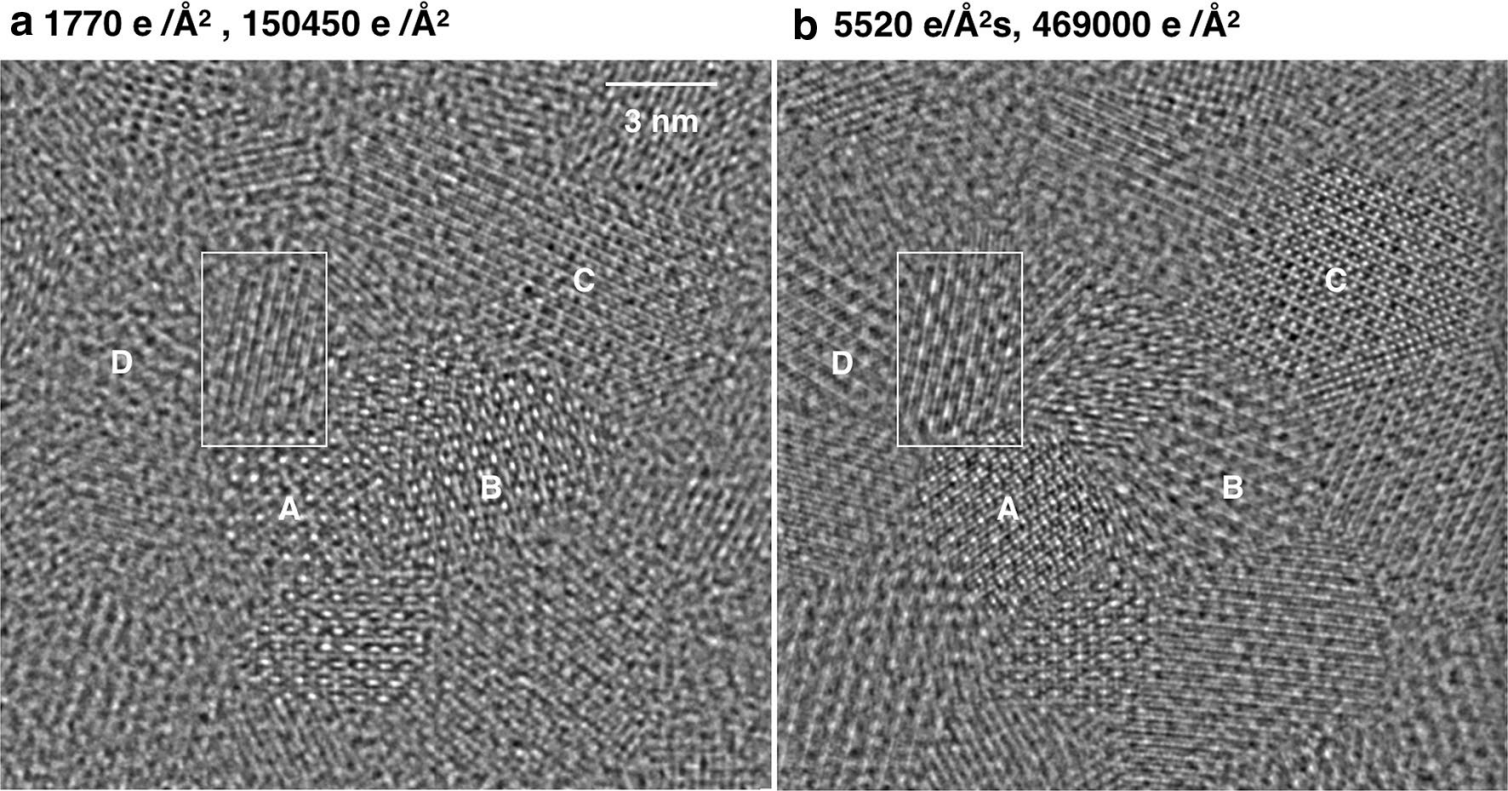
Electron beam-induced grain reorientation and growth in a polycrystalline Co_3_O_4_ film of ~4 nm thickness that was deposited by ALD on an electron transparent SiN membrane. The electron beam acceleration voltage is 300 kV. Dose rates and total doses are indicated. Reconstructed phase images are shown. A *box* marks a specific grain that helps relating both images to each other. Frames **a** and **b** list dose rates and the total electron dose for the series. Comparing frame **a** and **b** grains labeled *A* ([110 zone axis orientation) and *C* ([100] zone axis orientation) exhibit dominant grain growth because their zone axis pattern is almost maintained. Grains labeled *B* and *D* exhibit rotations, their respective zone axis pattern change

Since the frequency of atom displacements at surfaces increases with the beam current and peaks at medium acceleration voltages, beam-induced object alterations become much more visible if atoms are rapidly displaced from their lattice sites using high dose-rate conditions. Commonly, individual atoms are not instantly lost after a displacement event, but remain in surface proximity and contribute to object alterations by diffusion, as already reported for another material system [44, 45]. Before investigating single grains of the Co_3_O_4_ catalysts, it is instructive to consider a dense monolayer of ~4 nm large grains that form a continuous layer on a SiN membrane [38]. Figure 5 shows two reconstructed phase images from an identical sample area that is exposed to a large total electron dose of 150,000 and 469,000 e/Å^2^, successively. Our image reconstruction makes use of 50 individual frames from each series; it took 2.8 min of recording time to capture all images. A visual inspection of the images in Fig. 5 directly reveal that significant electron beam-induced grain growth and grain reorientation is stimulated, as specified in the figure caption. Such sintering processes of nanoparticles were studied by in situ plan-view transmission electron microscopy as early as 1998 [46] at a lower magnification by raising temperature to stimulate the observed transformations. There, it was pointed out that grain reorientations occur by grain boundary migration, grain rotation, and surface diffusion, which are exactly the processes that we can now observe at atomic resolution using the electron beam as a stimulus. It is remarkable that each image by itself gives the impression of being static and radiation resistant in spite of the fact that a strongly dynamic situation is created by the large beam currents. Certainly, time averaged structures are displayed in Fig. 5, but they hardly show any image blur, even at the chosen resolution of one Ångstrom. The illusion of a static situation is fostered because atom diffusion is fast compared to the image recording time and the evolution of grain growth is not arbitrary, but is linked to specific lattice sites.

**Fig. 6:**
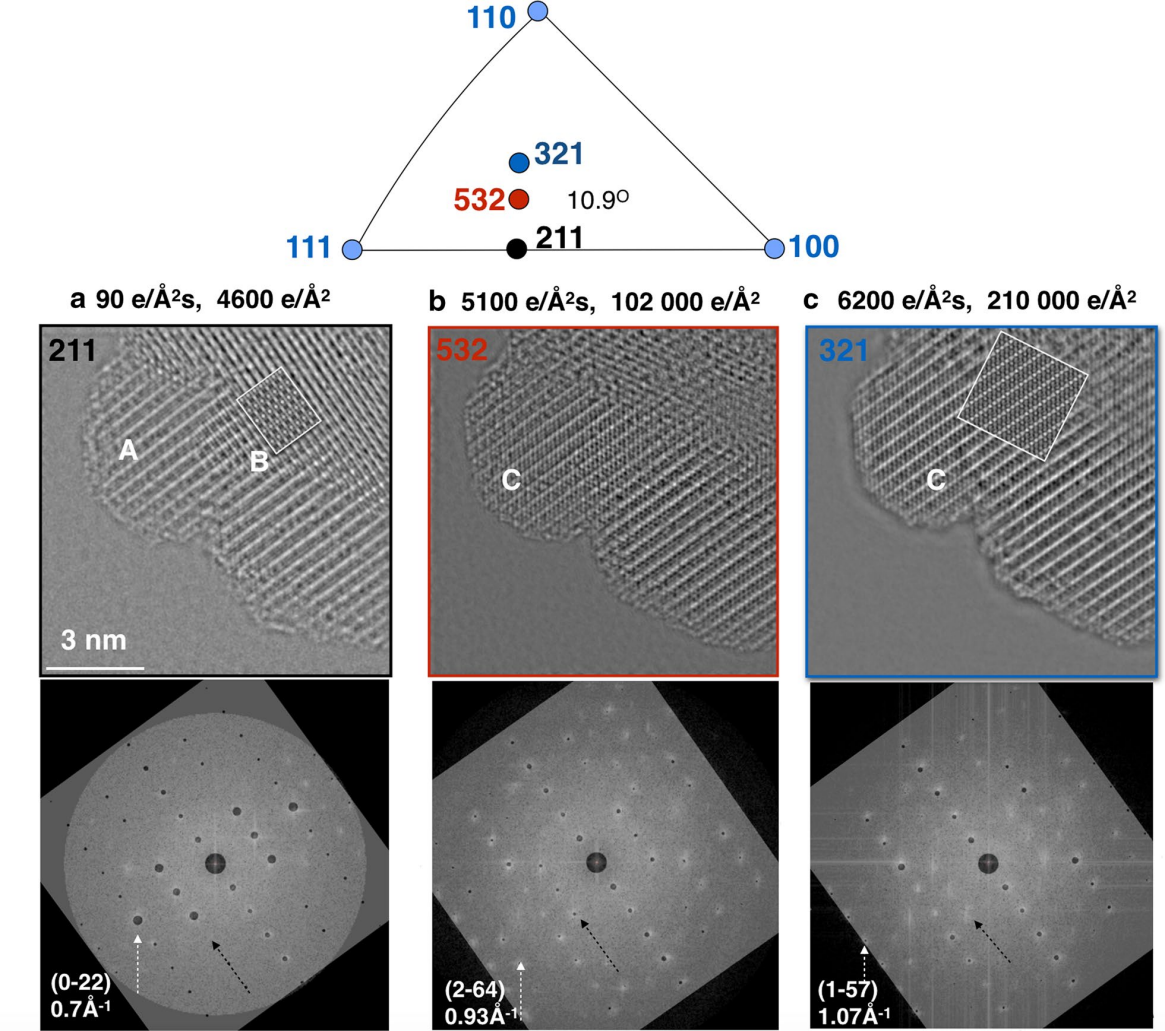
Electron beam-induced grain rotation of a single Co_3_O_4_ grain. The sample is prepared by surfactant-assisted solvothermal growth. The mid-voltage range electron beam acceleration is 80 kV. **a**–**c** show successively recorded phase images with dose rates and total doses as indicated. *A*, *B*, *C* highlight the presence of different crystal structures. It is seen that the [211] crystal orientation exhibit two distinctly different patterns *A*, *B*. *A* is an unknown atom configuration and *B* can be modeled by the expected atom configuration of a [211] oriented spinel Co_3_O_4_ grain as shown by the *inset* in Fig. 1a). *C* is the expected [321] pattern of the spinel Co_3_O_4_ as confirmed by the depicted image simulation, too. The local nano-diffraction patterns of the reconstructed wave functions are shown below, together with matched, calculated kinematic diffraction patterns that are used to identify the listed zone axes orientations. *Black arrows* point to specific diffraction spots that dominate in the [211] zone axis orientation but remain visible even in the [321] crystal orientation. On top, the standard stereographic projection of fcc crystal structure shows the suggested grain rotation of 10.9°

With these experimental results in mind, we now analyze electron beam-induced object alterations in the single Co_3_O_4_ particle shown in Fig. 6. Its genuine crystal structure is shown in Fig. 6a, which is recorded with a low dose rate of 90 e/Å^2^s and a total electron dose of 4600 e/Å^2^ that allows for the detection of single Co atoms but requires at least 2 O atoms to generate a suitable signal above noise. A coexistence of two image patterns A, B is pointed out in Fig. 6a. The image pattern B can be approximated by simulating a spinel structure of the [211] oriented Co_3_O_4_ particle. However, structure A can only be partly be explained in this manner for the following reason: upon prolonged exposure of the crystal to a more intense electron beam, both patterns A, B disappear and are replaced by the pattern C (Fig. 6b, c), which can be approximated by simulating a [321] grain orientation shown in Fig. 6c. In fact, all reflections of the nano-diffraction patterns in Fig. 6 can be reproduced by simulating the kinematic electron diffraction patterns of the spinel structure in [211], [531], and [321] zone axis orientations. This match of the simulations with the experiment is shown by insets in Fig. 6 and suggests a beam-induced grain rotation by 10.9 degrees that occurs as a result of an increased beam current. The geometry of the rotation is shown in the stereographic projection of Fig. 6. However, the different pole axes orientations do not strictly appear sequentially as one would expect if a grain rotates from a [211] to a [321] orientation. Instead, characteristic reflections coexist over the entire rotation range, such as the three dominant reflections from the [211] zone axis orientation that are pointed out by arrows. They should be absent from the [321] diffraction pattern, even if the excitation errors are large because the particles are small. Thus, the question arises if the observed crystal rotation is purely geometrical or if atom diffusion contributes, too, because it is strongly stimulated in high dose-rate conditions, as shown above.

**Fig. 7:**
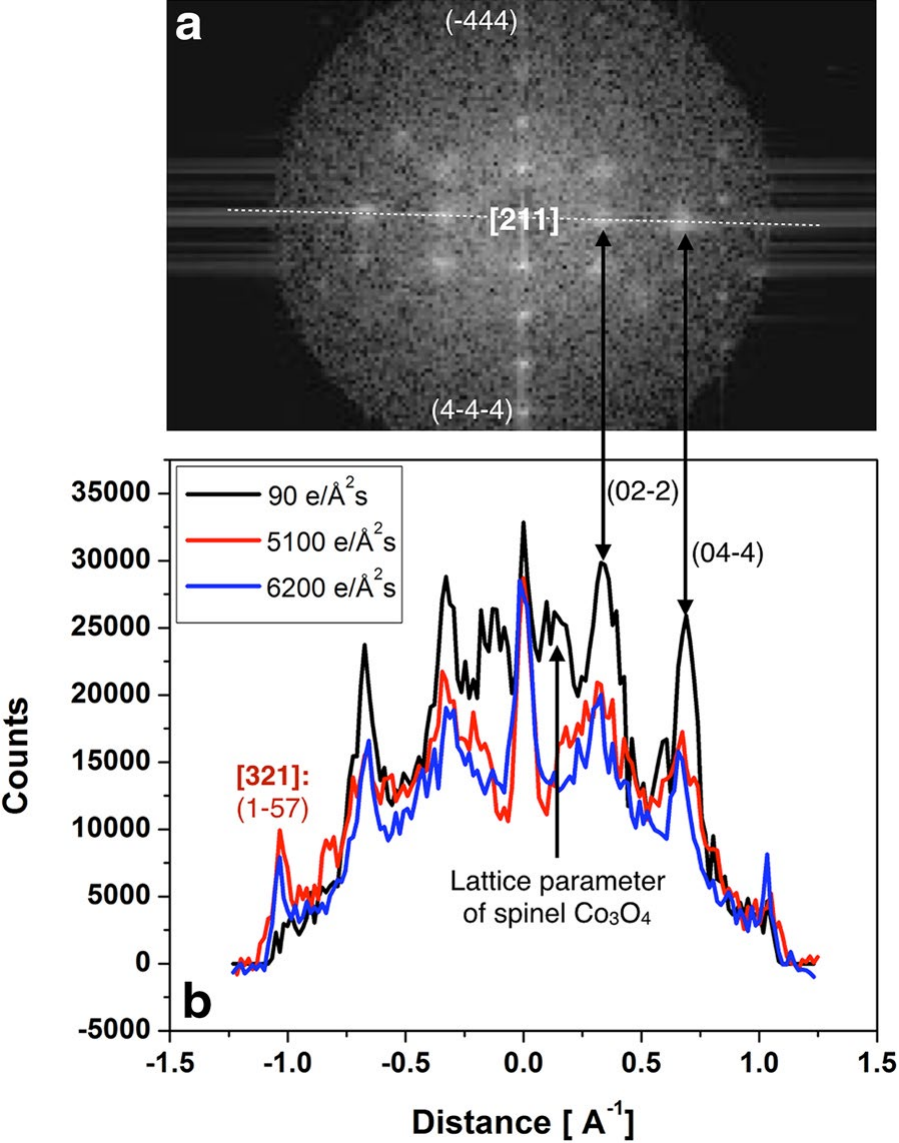
Electron beam-induced structure transformations in a single Co_3_O_4_ grain. **a** Indexed Fourier Transform of the [211] oriented grain of Fig. 6a. Note that the two image patterns A and B of Fig. 6a are hidden behind the same diffraction pattern. Line profiles are extracted along the* dotted line*. Note the significant broadening of the {022} and {044} diffraction spots if compared to the (4-4-4) diffraction spot, for example. **b** Line traces capturing the intensity of the indexed reflections of the Fourier transformed wave functions of Fig. 6a–c. Note the partial loss of intensity upon an increase of the dose rate

We address beam-induced atom diffusion in Fig. 7 by probing for rotation-induced intensity changes between the (0–22) and (0–44) reflections and their centro-symmetrically equivalent (02–2) and (04–4) reflections. This measurement is only meaningful because we consider the Fourier Transforms of a complex wave function, which is not forced to be centro-symmetric by mathematics. Reflection intensities are extracted along the dotted line in Fig. 7a. A visually equal intensity of the centro-symmetric diffraction pairs in this image characterize an exact [211] zone axis orientation, while the different intensities of the (−444) and (4–4–4) reflections capture a rotation of ~1° around the perpendicular crystal axis. In Fig. 7b, we compare quantitatively these line profiles from all three Fourier Transforms of Fig. 6a–c. A geometric crystal rotation by 10.9° must change the intensity ratio of centro-symmetric reflection spots drastically, while a constant reduction factor would point towards a changing volume fraction of a particular structural phase. Unexpectedly, both situations are observed simultaneously in Fig. 7b but concerning different sets of reflections: the relative intensities of the {220} and {440} reflections change their magnitude collectively to maintain their relative intensity closely, which excludes a large crystal rotation. On the other hand, different reflections emerge in Fig. 7b, such as the (1–57) reflection of the [321] crystal orientation because of the changing diffraction patterns that are shown in Fig. 6. This suggests the presence of a crystal rotation. Consequently, the measurement of Fig. 7b directly conflicts with a model of a beam-induced geometrical sample tilt, only.

Moreover, the measured crystal structure labeled A in Fig. 6a is incompatible with the simulated image of the Co_3_O_4_ spinel phase in [211] orientation that is present in a different area of the sample. Thus, the nano-diffraction pattern of Fig. 6a contains all reflections that are allowed to appear in the existing crystal symmetry of the spinel structure but the occupation of lattice sites with atoms must differ locally. This is a manifestation of the phase problem that we solve by reconstructing the electron exit wave function [41]. In Fig. 6c, the contribution of a [211] pattern to a [321] orientated crystal remains visible in reciprocal space but is reduced beyond recognition in real space, which cooperates with the partial loss of intensity upon an increase of the dose rate in Fig. 7b. In addition, the {220} and {440} reflections are significantly broadened if compared to other reflections, as pointed out in Fig. 7a.

This coexistence of different diffraction patterns over a substantial range of the stereographic projection, the simultaneous existence of different crystal structures in real space images with similar diffraction patterns, and the unusual broadening of specific reflections strongly suggest that the initial grain consists of a spinel structure at its core but may be covered by an external region of surface reconstructions. Electron beam-induced atom diffusion starts altering the crystal structure and orientation if the total electron dose significantly exceeds ~5000 e/Å^2^s and the electrons are delivered at a rate larger than 100 e/Å^2^s. Such a model can accommodate all experimental results including a grain rotation that is partly driven by atom diffusion and partly by a pure geometrical rotation.

Our measurements point out comprehensively that pristine Co_3_O_4_ structures, including their surfaces, can be reproducibly captured and analyzed in atomic resolution images with single atom sensitivity by keeping the dose rates low and using the smallest amount of electrons that are needed to detect single atoms. Large doses and rates will produce images of a seemingly static structure that, however, is a time average of beam-stimulated structure alterations where atomic resolution is maintained. Other detailed considerations are possible and exciting but lie beyond the scope of this paper.

## Conclusion and outlook

This contribution addresses electron beam-induced sample alterations in atomic resolution electron microscopy using acceleration voltages between 80 and 300 kV and beam currents that range from atto-Amperes/Å^2^ to pico-Amperes/Å^2^. The significance of their control is highlighted by investigating Co_3_O_4_ catalysts to show that acquisition of images from pristine catalytic surfaces can be accomplished at atomic resolution with single atom sensitivity. The contrast from single atoms is optimized by operating the TEAM0.5 microscope at suitably low voltage, by delivering electrons with rates below 100 e/Å^2^s, and by capturing large image series that provide the needed number of scattering events to create sufficient contrast from single atoms without causing damage. Exploiting the current technology and electrons accelerated by 80 kV, we estimate a S/N ratio of ~2 for the detection of one gold atom or one carbon atom if ~600 or 20,000 e/Å^2^ are delivered, respectively, in the bright field phase contrast imaging mode. Beam-induced object alterations primarily affect atom sites possessing lowered binding energies, which are typically present at surfaces, interfaces, defects, or in radiation-sensitive amorphous materials and soft matter where they consistently cause undesirable contrast variations [15, 47].

Electron beam-sample interactions cannot be described by strictly isolated knock-on events, where atoms are abruptly removed from the object without further impact. Instead, an increase of doses and rates stimulates physically meaningful processes such as atom diffusion, surface reconstructions, and sintering. Furthermore, they may trigger distinct relaxation pathways that otherwise can only be captured if the temperature is raised or additional charge is provided. In this manner, we find that Co_3_O_4_ polycrystalline films exhibit a strong tendency to sinter and that single grains can assume previously unknown reconstructions that extend from the crystal surfaces into the sub-surface region of the catalysts on a nanometer scale. Beam-induced crystal reorientations are found to be affected by diffusion-stimulated atom rearrangements within the unit cell of the material.

**Fig. 8:**
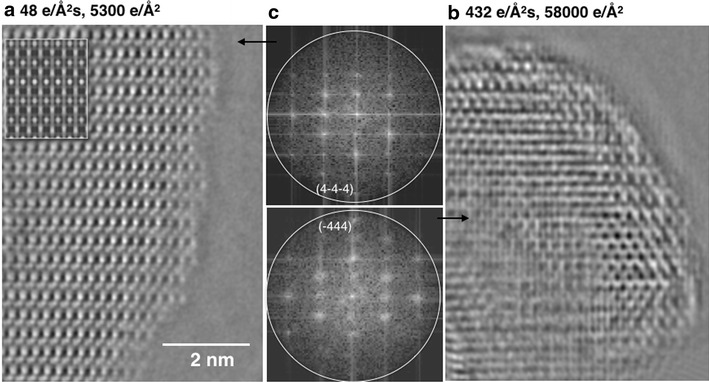
Impact of environments on the structure of Co_3_O_4_ catalysts, **a** the atomic structure of a [211] oriented Co_3_O_4_ catalyst with spinel structure fabricated by the high throughput printing process after an exposition to technical air at atmospheric pressure at 400 °C for 2 h. **b** Sample as in **a** but exposed in situ to 1 mbar of H_2_O vapor at 400 °C. **c** The related nano-diffraction patterns showing substantial peak broadening after the exposure to H_2_O vapor

Finally, it is pointed out that all our experiments are executed in the vacuum of the electron microscope, which is a high vacuum environment that, however, does not reflect the environment of functional catalysts, particularly when considering the impacts of temperature and pressure on structure. Figure 8 highlights the relevance of this aspect by comparing the structure of a spinel Co_3_O_4_ catalyst that was exposed to dry air with one that was exposed to water vapor. It is seen that an exposure to water vapor leads to a substantial disintegration of the spinel structure into a variety of locally different structures that all exhibit a diffuse [211] diffraction pattern, which is indistinguishable from the one shown in Fig. 7a. This dramatic structure alteration can now be shown to be genuine and it complements similar findings from the past [48]. Better understanding of such interactions, which are of considerable relevance to the real world, will require elevating environmental electron microscopy to an adequate level of resolution and sensitivity under atmospheric pressure and humidity. Finally, we stress the importance of not only recording diffraction patterns, but to also solving the phase problem with sub-Ångstrom precision because a rich range of otherwise unknown crystallographic nanostructures can be hidden behind a single diffraction pattern, as shown in this contribution.

## Footnotes

1 From Ref. [9], we estimate a total dose per image of ~30,000 e/Å^2^ from the given acquisition parameters of two sub-images that were recorded with a probe size of ∼50 pm, a beam current of 70 pA, a pixel size of 0.405 Å and a dwell time of 6 μs. For an image series of 62 images the total dose adds up to 1,900,000 e/Å^2^. A S/N ratio of 2 for the detection of a single W atom can be estimated by extracting line profiles from the supplementary Figure 2, the pyramidal shape of the needle, which makes it roughly as wide as it is deep, and assuming that noise is dominated by the dark current of the detector since the electron counts are high.

2 The total dose of ~90,000 e/Å^2^ in Ref. [7] can be estimated from the average number of counts = 3500 as shown in the supplementary Figure 1, a typical factor of 0.5 for CCD cameras to convert photon counts to electron counts at 300 kV, a given exposure time of 0.5 s and typical sampling rates of 0.2 Å/pixel at high resolution.
